# Clinical and biochemical characteristics of diabetic ketoacidosis in adults with type 1 or type 2 diabetes at a tertiary hospital in the United Arab Emirates

**DOI:** 10.3389/fcdhc.2022.918253

**Published:** 2022-08-08

**Authors:** Raya Almazrouei, Amatur Rahman Siddiqua, Mouza Alnuaimi, Saif Al-Shamsi, Romona Govender

**Affiliations:** ^1^ Department of Endocrinology, Tawam Hospital, Al Ain, United Arab Emirates; ^2^ Internal Medicine Department, College of Medicine and Health Sciences, United Arab Emirates University, Al Ain, United Arab Emirates; ^3^ Internal Medicine Department, Tawam Hospital, Al Ain, United Arab Emirates; ^4^ Family Medicine Department, College of Medicine and Health Sciences, United Arab Emirates University, Al Ain, United Arab Emirates

**Keywords:** type 1 diabetes mellitus, type 2 diabetes (T2D), diabetic ketoacidosis, diabetes ketoacidosis (DKA), diabetes emergencies

## Abstract

**Background:**

Diabetes ketoacidosis (DKA) is a well-known acute complication of diabetes. This study aims to describe the sociodemographic, clinical, and biochemical characteristics of adult patients with different diabetes types and DKA severities attending a tertiary hospital in the UAE.

**Methods:**

We retrospectively extracted sociodemographic, clinical, and laboratory data from the electronic medical records of 220 adult patients with DKA admitted to Tawam Hospital between January 2017 and October 2020.

**Results**

The average age was 30.6 ± 16.6 years of whom 54.5% were women, 77.7% were UAE nationals and 77.9% were Type 1 diabetes (T1DM). 12.7% were newly diagnosed with diabetes. Treatment noncompliance (31.4%), and infection (26.4%) were the main precipitating factors. Most patients presented with moderate DKA (50.9%). Compared to T1DM, patients with Type 2 diabetes (T2DM) were older (53.6 vs 23.9 years, p < 0.001), had longer hospital stay (12.1 days vs 4.1 days, p < 0.001), had more complications (52.1%, vs 18.9% p <0.001), and a higher mortality rate (6.3% vs 0.6%, p = 0.035). Patients with severe DKA had a shorter diabetes duration compared to mild and moderate DKA (5.7 vs 11.0 vs 11.7 years, respectively, p = 0.007), while complications were significantly lower in the mild group compared to both the moderate and severe groups (11.6% vs 32.1% vs 33.3%, respectively).

**Conclusion:**

The risk of DKA is higher for patients with T1DM than for those with T2DM. The clinical characteristics and outcomes of patients with T2DM differ from those with T1DM highlighting the importance of educating all patients about DKA.

## Introduction

Diabetes mellitus (DM) is a metabolic disease characterized by hyperglycemia due to the impairment in insulin secretion or insulin resistance. The two main types of DM are type 1 diabetes (T1DM) and type 2 diabetes (T2DM). While T1DM is more common in the young age group (childhood and adolescence), T2DM is encountered more with increasing age. In the Middle East and North Africa (MENA) region, the 2021 prevalence of diabetes is 16.2% (8.5–18.3) and the United Arab Emirates (UAE) belongs to this region (1). The prevalence of diabetes in the UAE is alarming with age-adjusted comparative prevalence of diabetes of 16.4% in 2021, which is projected to increase to 18.1% by 2045 (2). No solid data are available about T1DM prevalence in UAE.

DM, together with its macrovascular and microvascular complications, is associated with an increased risk of morbidity and mortality. Diabetic ketoacidosis (DKA), described as one of the most severe acute life-threatening complications, has an overall mortality rate ranging from 0.2% to 2%, with persons at the highest end of the range residing in developing countries (3). DKA is characterized by biochemical abnormalities including hyperglycemia, ketonemia, and metabolic acidosis and is usually diagnosed in patients with T1DM (incidence, 50–100 episodes per 1,000 patients) but is a less frequent complication of T2DM with an incidence rate of 4.6–8 episodes per 1,000 patients (4). A local single-center retrospective study showed that T1DM constitutes 61% of DKA admissions compared to 27.1% for T2DM (5), and as expected, those with T2DM are older (6). The most common precipitating causes of DKA in most studies were nonadherence to therapy, infections, and undiagnosed diabetes with data from the UAE reporting 31.4%, 22.7%, and 12%, respectively (5, 7, 8). Only a few studies have explored the clinical and biochemical characteristics of DKA in the MENA region, including in the UAE. As far as we are aware, none of these studies have assessed DKA characteristics according to the diabetes type and the degree of DKA severity. Thus, we aimed to describe the clinical and biochemical characteristics, and the severity of DKA among adults with T1DM or T2DM admitted to a tertiary care hospital in the UAE.

## Methods

We reviewed records of all patients aged 16 years (local cutoff age for adult service) and older admitted with DKA to Tawam Hospital (Al Ain, UAE) from January 2017 to October 2020. We retrieved the records using ICD 10 CM codes (E10.10, E10.11, E13.10, and E13.11) for DKA episodes. Prior to April 2020, Tawam was the designated hospital for Emirati nationals, employees, and a few other medical insurance holders, while non-Emirati nationals and other individuals with different medical insurance types were seen at another public hospital. The protocol used at Tawam Hospital for DKA diagnosis and treatment is derived from international guidelines (9, 10). Using the American Diabetes Association (ADA) criteria, the DKA severity was classified according to the pH value as mild (pH 7.3–7.25), moderate (pH 7.24–7), or severe (pH <7) (9). The diabetes types were diagnosed on the basis of history and/or autoantibodies statuses. We extracted data on patients’ sociodemographic characteristics, initial clinical and chemical findings on admission, and DKA outcomes from the electronic medical records. We also retrieved data on comorbidities and complications from the medical notes and confirmed them using the list of persistent and acute problems in the electronic medical records. Finally, for every case, we recorded the hemoglobin A1c (HbA1c) value on admission or within the preceding 3 months. The Tawam Human Research Ethics Committee approved the study (MF2058-2022-826) and waived the requirement for consent due to the retrospective nature of the study, the anonymity of the data collection, and the lack of interventions.

### Statistical analyses

We described categorical variables as proportions and continuous variables as means ± standard deviations (SDs). The means between two groups were compared using the Student’s *t*-test, and the means among more than three groups were compared using the one-way ANOVA and the least significant differences test. In addition, we applied Fisher’s exact test (two-tailed) to compare proportions. All results were performed using SPSS for Windows v28.0 (IBM, Armonk, NY, USA). *p*-values < 0.05 (two-sided) were considered as statistically significant.

## Results

**Figure 1:**
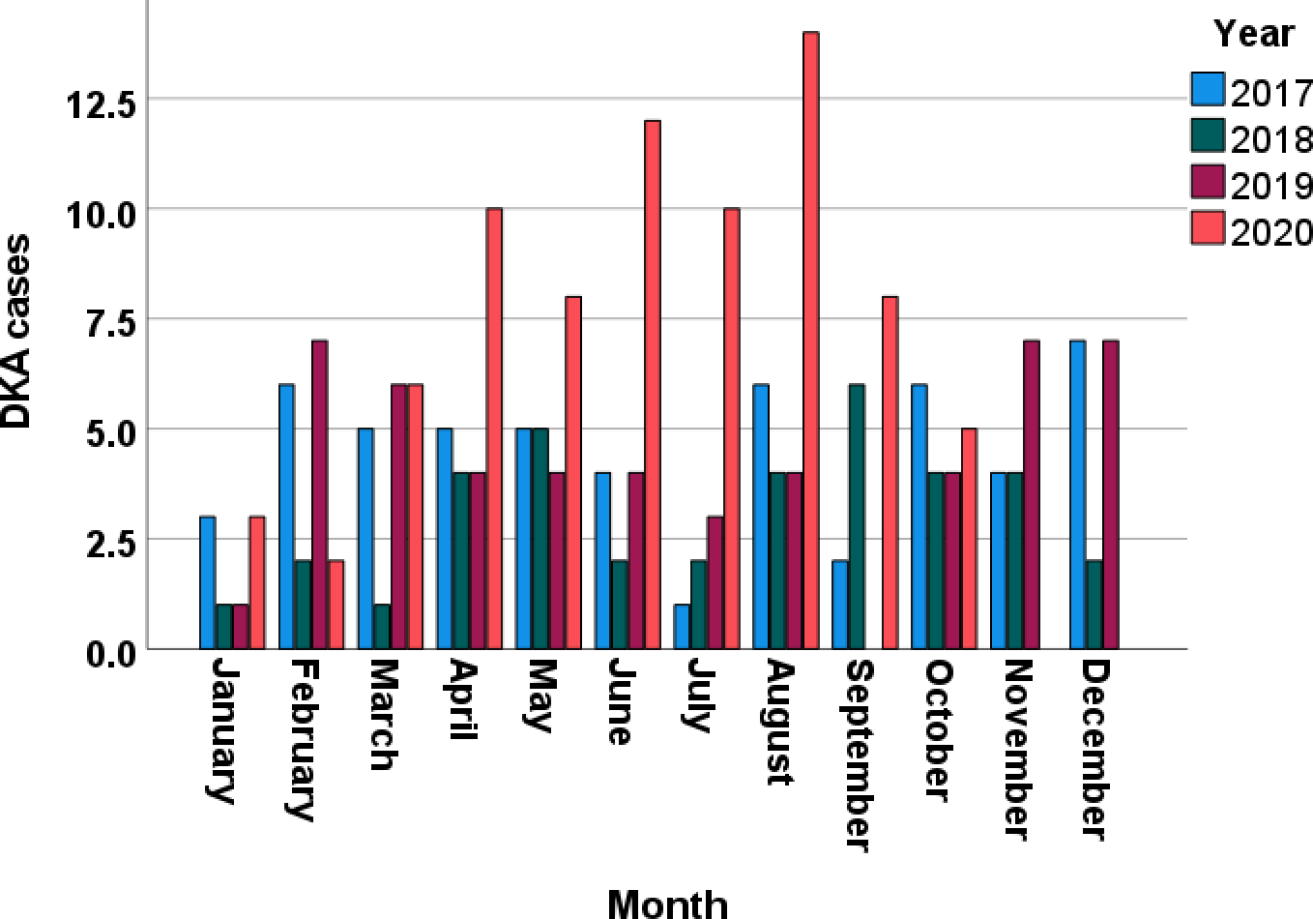
DKA cases per month between January 2017 and October 2020.

**Figure 2:**
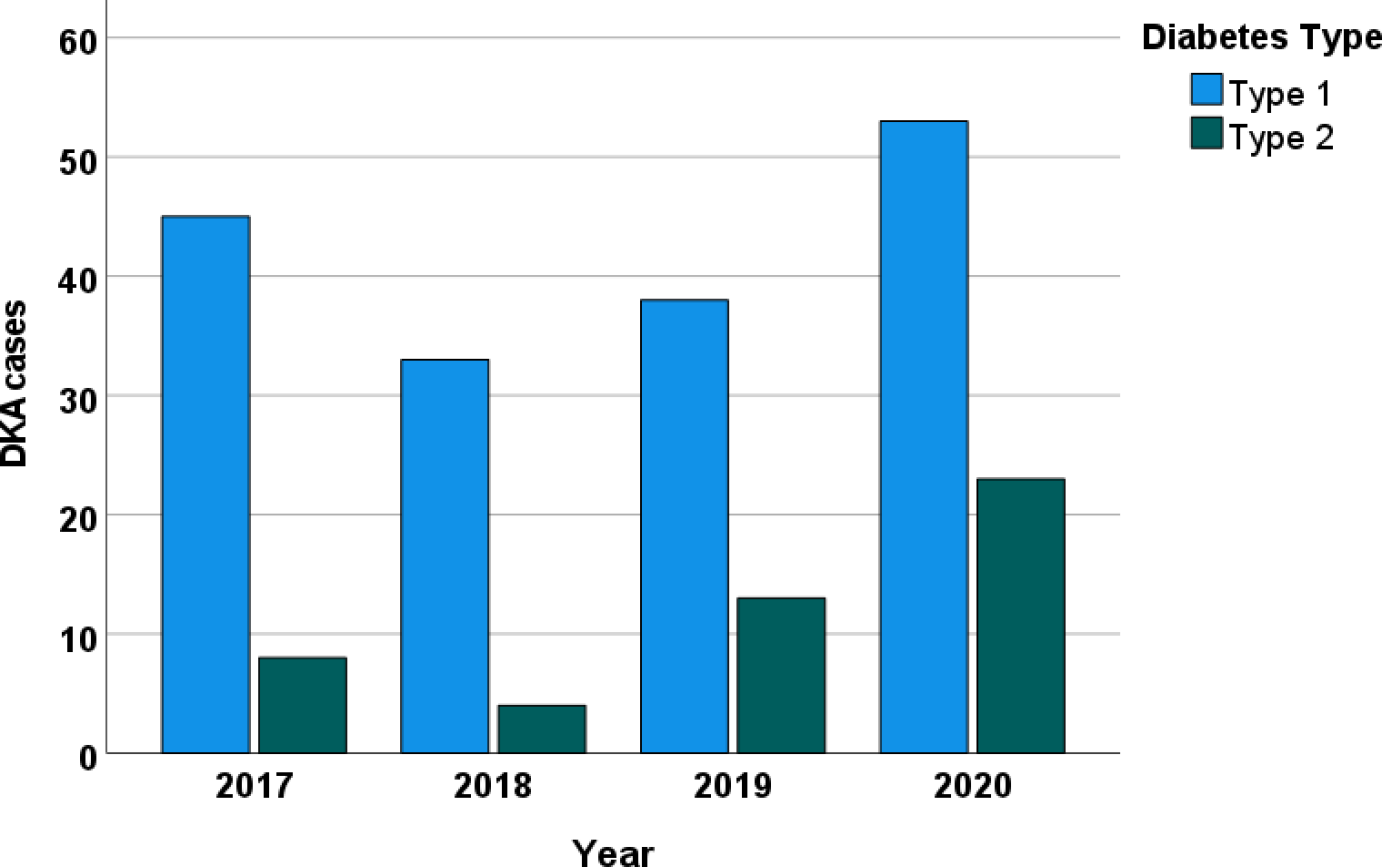
Total number of DKA cases per year over the study period.

We collected data from 220 consecutive patients with DKA at Tawam Hospital. **Table 1** lists the patients’ sociodemographic characteristics upon admission. The average age was 30.6 ± 16.6 years (range, 16–87 years); 54.5% (120/220) of patients were women; 77.7% (171/220) were Emirati nationals. Overall, 77.9% (169/217) of the patients had T1DM, and 22.1% (48/217) had T2DM. On admission, 12.7% (28/220) of the patients were newly diagnosed as having diabetes. The mean duration of diabetes in patients diagnosed prior to admission was 10.4 ± 9.3 years, and 62.3% (134/215) had had previous DKA admissions. The proportion of patients with a previous history of DKA was significantly lower in non-Emirati individuals than in Emirati nationals (28.3% vs. 71.6, *p* < 0.001). **Figure 1** depicts the increase in monthly DKA admissions after April 2020 compared to matched months over the previous years due to service reallocations during the SARS-CoV-2 pandemic. **Figure 2** shows the increase in DKA episodes in individuals with T2DM over the study period.

**Table 1:** Sociodemographic characteristics of the DKA cases at admission.

Variable	Total (*n* = 220)
**Age (years), mean ± SD**	30.6 ± 16.6
**Sex, *n* (%)**	
Female Male	120 (54.5) 100 (45.5)
**Nationality, *n* (%)**	
Emirati Arab South Asian Other	171 (77.7) 25 (11.4) 18 (8.2) 6 (2.7)
**Insurance status (yes), *n* (%)**	188 (85.5)
**Job status, *n* (%) (*n* = 204)**	
Student Employed Unemployed	95 (46.6) 63 (30.9) 46 (22.5)
**Diabetes type, *n* (%) (*n* = 217)**	
Type 1 Type 2	169 (77.9) 48 (22.1)
**Newly diagnoses, *n* (%)**	28 (12.7)
**Diabetes duration (years), mean ± SD** **(*n* = 171)**	10.4 ± 9.3
**Baseline medication**	
Metformin, *n* (%)* Sulfonylurea, *n* (%) DDP4i, *n* (%) SGL2i, *n* (%) GLP-1 agonist, *n* (%) Insulin, *n* (%)	25 (11.4) 10 (4.5) 16 (7.3) 10 (4.5) 5 (2.3) 177 (80.5)
**Insulin delivery method use, *n* (%)**	
MDI CSII	151 (68.6) 26 (11.8)
**Previous DKA, *n* (%) (*n* = 215)**	134 (62.3)
**Comorbidities**	
Microvascular complications, *n* (%) Macrovascular complications, *n* (%) CKD, *n* (%) Active Cancer, *n* (%)	50 (22.7) 8 (3.6) 17 (7.7) 6 (2.7)

SD, standard deviation; DDP4i, dipeptidyl peptidase-4 inhibitors; SGL2i, sodium/glucose cotransporter-2 inhibitors; GLP-1, glucagon-like peptide-1; MDI, multiple daily doses of insulin; CSII, continuous subcutaneous insulin infusion; CKD, chronic kidney disease. *Only 24 out of 48 T2DM were on metformin for possible uncollected reasons including intolerance, CKD, or patient self-option to reduce oral tablets if being on insulin.

The initial clinical and biochemical findings recorded in DKA admissions are described in **Table 2**. The top two precipitating factors were treatment noncompliance (31.4%), and infections (26.4%; mostly respiratory and urinary tract infections). We found 10 patients with T2DM and SGLT2 (sodium/glucose cotransporter 2) inhibitor-associated DKA. On admission, the mean random serum glucose level was 24.0 ± 8.0 mmol/L and the mean HbA1c level was 11.0 ± 2.7%. **Table 3** presents the DKA outcomes in this cohort. The mean length of hospital stay was 5.8 ± 8.8 days, and the total in-hospital mortality rate among patients was 4 (1.8%). We found 31.4% (69/220) mild, 50.9% (112/220) moderate, and 17.7% (39/220) severe DKA cases.

**Table 2:** Initial clinical and biochemical findings in patients presenting with DKA.

Variable	Total (*n* = 220)
**Presenting symptoms**	
Nausea and vomiting, *n* (%) Abdominal pain, *n* (%) Shortness of breath, *n* (%) Polyuria and polydipsia, *n* (%) Weight loss, *n* (%) Impaired LOC, *n* (%) Fever, *n* (%) Newly diagnoses, *n* (%)	156 (70.9) 125 (56.8) 31 (14.1) 37 (16.8) 11 (5.0) 31 (14.1) 33 (15.0) 28 (12.7)
**Precipitating factors, *n* (%)**	
Newly diagnosed alone Insulin omission/noncompliance Infection alone Drug-induced alone Trauma Post-surgery Indeterminate Pump malfunction Combination of above	23 (10.5) 69 (31.4) 58 (26.4) 5 (2.3) 1 (0.5) 5 (2.3) 23 (10.5) 9 (4.1) 27 (12.3)
**Infection types ( ± combination), *n* (%)**	
Upper respiratory tract infection Urinary tract infection Pneumonia Gastroenteritis Skin and soft tissue Sepsis with bacteremia Dental abscess/caries	32 (14.5) 19 (8.6) 13 (5.9) 5 (2.3) 3 (1.4) 7 (3.2) 3 (1.4)
**Drug-induced ( ± combination), *n* (%)**	
SGLT2I Steroid GLP-1	10 (4.5) 4 (1.8) 1 (0.5)
**Vital signs at presentation**	
Weight (kg) ± SD BMI (kg/m²) ± SD (*n* = 212) SBP (mmHg) ± SD Heart rate (beats/minute) ± SD Respiratory rate (breaths/minute) ± SD	63.6 ± 18.9 24.1 ± 6.6 123.0 ± 22.8 113.6 ± 21.7 22.7 ± 6.4
**Initial venous blood gas results**	
pH (unitless) ± SD HCO_3_ (mmol/L) ± SD Glucose (mmol/L) ± SD	7.1 ± 0.1 10.2 ± 4.9 23.0 ± 7.2
**Initial blood test results**	
Na (mmol/L) ± SD K (mmol/L) ± SD CL (mmol/L) ± SD Urea (mmol/L) ± SD HCO_3_ (mmol/L) ± SD Anion gap (mmol/L) ± SD Serum glucose (mmol/L) ± SD HbA1c (%) ± SD (*n* = 208)	132.9 ± 4.9 4.4 ± 0.9 97.5 ± 7.4 7.0 ± 6.8 9.7 ± 4.8 25.6 ± 7.3 24.0 ± 8.0 11.0 ± 2.7
**Urine ketones, *n* (%) (*n* = 211)**	
+1 +2 +3 +4 +5	17 (7.7) 26 (11.8) 152 (69.1) 13 (5.9) 3 (1.4)

LOC, loss of consciousness; SD, standard deviation; SGL2i, sodium/glucose cotransporter-2 inhibitors; GLP-1, glucagon-like peptide-1; BMI, body mass index; SBP, systolic blood pressure; HbA1c, hemoglobin A1c.

**Table 3:** Diabetes ketoacidosis outcomes.

Variable	Total (*n* = 220)
**ICU/HDU admission**, *n* (%) **Time to DKA resolution**, (h) ± SD (*n* = 215) **Length of hospital stay**, (days) ± SD **In-hospital mortality**, *n* (%) **Mechanical ventilation**, *n* (%)	163 (74.1) 19.1 ± 15.9 5.8 ± 8.8 4 (1.8) 11 (5.0)
**Complications, *n* (%)**	
None Pulmonary edema Hypokalemia DKA relapse MI/Stroke Venous thrombosis Pancreatitis More than 1 complication (combo)	163 (74.1) 4 (1.8) 37 (16.8) 4 (1.8) 4 (1.8) 1 (0.5) 4 (1.8) 3 (1.4)
**DKA severity, *n* (%)**	
Mild Moderate Severe	69 (31.4) 112 (50.9) 39 (17.7)

ICU, intensive care unit; HDU, high dependency unit; DKA, diabetic ketoacidosis; MI, myocardial infarction.

The clinical characteristics of the diabetes type groups in the study population are shown in **Table 4**. Patients with T1DM were significantly younger than those with T2DM (23.9 vs. 53.6 years, *p* < 0.001). Among Emirati patients with DKA, most of them had T1DM, whereas T2DM was more common among the non-Emirati patients with DKA. Most patients with T1DM (76.4%) had a previous history of DKA, as compared to only 17% of the patients with T2DM (*p* < 0.001). The proportion of patients with microvascular and macrovascular complications, and chronic kidney disease (CKD), was higher in the T2DM group, and most patients requiring ICU admission belonged to the T2DM group as well. The average lengths of stay in hospital were 12.1 days for patients with T2DM and 4.1 days for patients with T1DM (*p* < 0.001). The mortality rate was highest in the T2DM group at 6.3%, whereas the recorded mortality rate in patients with T1DM was 0.6% (*p* = 0.035). The proportions of complications observed in patients with T1DM (18.9%) and T2DM (52.1%) differed significantly (*p* < 0.001).

**Table 4:** Clinical characteristics of patients according to diabetes type (*n* = 217).

Characteristic	Type 1 (*n* = 169)	Type 2 (*n* = 48)	*p*-value
Age (years), mean ± SD	23.9 ± 7.7	53.6 ± 18.9	<0.001
**Sex, *n* (%)**			
Female Male	96 (56.8) 73 (43.2)	24 (50.0) 24 (50.0)	0.416
**New diagnoses, *n* (%)**	20 (11.8)	7 (14.6)	0.623
**Nationality*, n* (%)**			
Emirati Non-Emirati	140 (82.8) 29 (17.2)	30 (62.5) 18 (37.5)	0.005
**Diabetes duration** (years), mean ± SD (*n* = 170)	9.3 ± 6.4	15.4 ± 16.2	0.049
**Baseline medication**			
Metformin, *n* (%)* Sulfonylurea, *n* (%) DDP4i, *n* (%)* SGL2i, *n* (%) GLP-1 agonist, *n* (%) Insulin, *n* (%)	1 (0.6) 0 (0.0) 1 (0.6) 0 (0.0) 0 (0.0) 149 (88.2)	24 (50.0) 10 (20.8) 15 (31.3) 10 (20.8) 5 (10.4) 26 (54.2)	<0.01 <0.01 <0.01 <0.01 <0.01 <0.01
**Previous DKA, *n* (%) (*n* = 212)**	126 (76.4)	8 (17.0)	<0.001
**Comorbidities**			
Microvascular complications, *n* (%) Macrovascular complications, *n* (%) CKD, *n* (%) Active Cancer, *n* (%)	32 (18.9) 0 (0.0) 6 (3.6) 1 (0.6)	18 (37.5) 8 (16.7) 11 (22.9) 5 (0.4)	0.011 <0.001 <0.001 0.002
**Initial blood test results**			
HbA1c (%) ± SD (*n* = 206) Serum glucose (mmol/L) ± SD Serum Na (mmol/L) ± SD	11.2 ± 2.8 24.2 ± 7.9 132.7 ± 4.6	10.2 ± 2.3 23.0 ± 7.6 133.5 ± 6.2	0.018 0.345 0.378
**DKA severity, *n* (%)**			
Mild Moderate Severe	60 (35.5) 79 (46.7) 30 (17.8)	9 (18.8) 31 (64.6) 8 (16.7)	0.060
**Outcomes of diabetes ketoacidosis**			
ICU admission, *n* (%) Time to DKA resolution, (h) ± SD (*n* = 212) Length of hospital stay, (days) ± SD In-hospital mortality, *n* (%) Mechanical ventilation, *n* (%) Complications, *n* (%)	119 (70.4) 17.7 ± 14.4 4.1 ± 4.5 1 (0.6) 2 (1.2) 32 (18.9)	41 (85.4) 24.1 ± 19.7 12.1 ± 15.3 3 (6.3) 9 (18.8) 25 (52.1)	0.041 0.040 <0.001 0.035 <0.001 <0.001

SD, standard deviation; DKA, diabetic ketoacidosis; HbA1c, hemoglobin A1c; CKD, chronic kidney disease; ICU, intensive care unit. *Same patient with late autoimmune diabetes in adults.

**Table 5** shows the comparison in clinical characteristics among the mild, moderate, and severe groups of patients with DKA. Most patients with mild DKA were women, whereas most in the moderate group were men. We found a shorter duration of diabetes in patients with severe DKA than in those with mild and moderate DKA (5.7 vs. 11.0 vs. 11.7 years, respectively, *p* = 0.007). Interestingly, the mild DKA group had a higher proportion of patients with previous DKA episodes than the moderate DKA group (72.1% vs. 53.7%).

**Table 5:** Clinical features of patients with mild, moderate, and severe DKA.

Characteristic	Mild (*n* = 69)	Moderate (*n* = 112)	Severe (*n* = 39)	*p*-value
Age (years), mean ± SD	28.8 ± 13.7	32.9 ± 18.6	27.5 ± 14.3	0.117
**Sex, *n* (%)**				
Female Male	46 (66.7) 23 (33.3)	54 (48.2) 58 (51.8)	20 (51.3) 19 (48.7)	0.047^a^
**Nationality, *n* (%)**				
Emirati Non-Emirati	57 (82.6) 12 (17.4)	85 (75.9) 27 (24.1)	29 (74.4) 10 (25.6)	0.479
**Insurance status (yes), *n* (%)**	63 (91.3)	93 (83.0)	32 (82.1)	0.244
**Job status, *n* (%) (*n* = 204)**				
Student Employed Unemployed	31 (48.4) 24 (37.5) 9 (14.1)	43 (41.7) 29 (28.2) 31 (30.1)	21 (56.8) 10 (27.0) 6 (16.2)	0.099
**Diabetes type, *n* (%) (*n* = 217)**				
Type 1 Type 2	60 (87.0) 9 (13.0)	79 (71.8) 31 (28.2)	30 (78.9) 8 (21.1)	0.060
**Diabetes duration** (years), mean ± SD (*n* = 171)	11.0 ± 7.2	11.7 ± 11.3	5.7 ± 3.6	0.007^bc^
**Previous DKA**, *n* (%) (*n* = 215)	49 (72.1)	58 (53.7)	27 (69.2)	0.033^a^
**Presenting symptoms**				
Nausea and vomiting, *n* (%) Abdominal pain, *n* (%) Shortness of breath, *n* (%) Polyuria and polydipsia, *n* (%) Weight loss, *n* (%) Impaired loss of consciousness, n (%) Fever, *n* (%) Newly diagnoses, *n* (%)	48 (69.6) 39 (56.5) 9 (13.0) 13 (18.8) 4 (5.8) 2 (2.9) 15 (21.7) 10 (14.5)	80 (71.4) 62 (55.4) 8 (7.1) 19 (17.0) 6 (5.4) 15 (13.4) 17 (15.2) 14 (12.5)	28 (71.8) 24 (61.5) 14 (35.9) 5 (12.8) 1 (2.6) 14 (35.9) 1 (2.6) 4 (10.3)	0.980 0.827 <0.001^bc^ 0.741 0.843 <0.001^bc^ 0.017^b^ 0.892
**Vital signs at presentation**				
Weight (kg) ± SD BMI (kg/m²) ± SD (*n* = 212) SBP (mmHg) ± SD Heart rate (beats per minute) ± SD Respiratory rate (breaths per minute) ± SD	64.2 ± 20.2 24.9 ± 7.2 119.6 ± 23.5 106.0 ± 23.5 21.0 ± 7.0	64.0 ± 18.7 24.0 ± 6.5 122.4 ± 21.6 113.9 ± 20.3 21.7 ± 4.5	61.3 ± 17.4 23.1 ± 5.9 130.6 ± 23.7 126.5 ± 15.6 28.3 ± 6.9	0.705 0.384 0.049^bc^ <0.001^abc^ <0.001^bc^
**Initial blood test results**				
Na (mmol/L) ± SD K (mmol/L) ± SD CL (mmol/L) ± SD Urea (mmol/L) ± SD HCO_3_ (mmol/L) ± SD Anion gap (mmol/L) ± SD Serum glucose (mmol/L) ± SD HbA1c (%) ± SD (*n* = 208)	132.7 ± 3.7 4.1 ± 0.6 97.3 ± 6.4 5.4 ± 4.2 14.4 ± 3.1 21.1 ± 5.4 23.3 ± 8.8 10.6 ± 2.9	133.3 ± 5.2 4.5 ± 0.9 97.8 ± 7.0 8.3 ± 8.7 8.8 ± 3.6 26.7 ± 7.2 23.7 ± 7.6 10.7 ± 2.4	131.7 ± 5.9 4.7 ± 1.0 97.3 ± 9.9 6.1 ± 3.0 4.3 ± 1.6 30.2 ± 6.3 26.2 ± 7.1 12.6 ± 2.7	0.201 0.002^ab^ 0.864 0.016^a^ <0.001^abc^ <0.001^abc^ 0.166 <0.001^bc^
**Outcomes of diabetes ketoacidosis**				
ICU admission, *n* (%) Time to DKA resolution, (h) ± SD (*n* = 215) Length of hospital stay, (days) ± SD In-hospital mortality, *n* (%) Mechanical ventilation, *n* (%) Complications, *n* (%)	30 (43.5) 11.1 ± 9.9 4.4 ± 3.7 0 (0.0) 1 (1.4) 8 (11.6)	94 (83.9) 21.1 ± 16.5 7.1 ± 11.5 4 (3.6) 8 (7.1) 36 (32.1)	39 (100.0) 28.0 ± 16.7 4.7 ± 4.7 0 (0.0) 2 (5.1) 13 (33.3)	<0.001^abc^ <0.001^abc^ 0.086^a^ 0.303 0.225 0.003^ab^

SD, standard deviation; DKA, diabetic ketoacidosis; BMI, body mass index; SBP, systolic blood pressure; HbA1c, hemoglobin A1c; ICU, intensive care unit.

mild group vs. moderate group, ^a^ (p < 0.05), mild group vs. severe group, ^b^ (p < 0.05), moderate group vs. severe group, ^c^ (p < 0.05).

In subgroup analysis stratified by sex, among the women with T1DM, the largest DKA group comprised mild cases when compared to the moderate and severe groups (91.3 vs. 68.5 vs. 85%, respectively, *p* = 0.014), whereas in men, all groups had similar (percentages).

As expected, the severe DKA group had a significantly higher proportion of patients presenting with shortness of breath, impaired level of consciousness (LOC), and ICU (intensive care unit) admissions than the mild and moderate groups. In addition, systolic blood pressure, heart rate, respiratory rate, anion gap, and HbA1c values were higher, and the time to DKA resolution was significantly longer in patients with severe DKA than in those with mild or moderate DKA.

The mean length of hospital stay was significantly longer in the moderate group than in the mild group (7.1 vs. 4.4 days), while compared to both the moderate and severe groups, the proportion of patients with complications was significantly lower in the mild group (32.1% vs. 33.3% vs. 11.6%, respectively).

## Discussion

Traditionally, DKA has been a characteristic complication of T1DM (9) and was once a hallmark for differentiating between cases of T1DM and T2DM. However, with 90%–95% of patients diagnosed with T2DM (11) and with DKA occasionally associated with T2DM, we therefore reviewed DKA hospital admissions for patients with both T1DM and T2DM. In this study, 77.9% of the patients admitted with DKA were patients with T1DM; however, we noted clinical and biochemical differences in DKA between the patients admitted with T1DM and those admitted with T2DM. Patients with T2DM admitted with DKA were older compared to patients with T1DM with longer disease duration and higher comorbidities. Most patients with T2DM were admitted with moderate DKA (64.6%) and 85.4% required ICU admission. We also found statistically significant differences in the outcomes of DKA according to the patients’ DM type with longer hospital stay, recovery time, and higher in-hospital mortality rate at 6.3% (3/48) in patients with T2DM compared to T1DM. These findings demonstrate the importance of alerting physicians to early recognition of DKA in at-risk patients with T2DM.

DKA accounts for 14% of the hospital admissions for patients with DM worldwide (3). Almost one-quarter of the DKA admissions in this study were for patients with T2DM, and we observed a steady increase in DKA admissions for individuals with T2DM from 2017 to 2020. These findings are supported by other studies (5, 12), whereas results from Saudi Arabia (a country within the MENA region) tended to be much lower at 7% (13), and about the same time, a study in Malaysia reported that at least half of their DKA admissions (51.5%) were patients with T2DM (13). This confirms the existence of epidemiological variability by country (13–16), and this may be reflective of the prevalence of diabetes in that population (17). The reasons for this variability are multifactorial and multidimensional (18), and they may be influenced by socioeconomic conditions, ethnicity, health systems, body mass index (lower BMI), infections, and delayed management (19). In our cohort, the highest proportion of T2DM patients with DKA was encountered in the year 2020. This is likely explained by the service redistribution during the COVID-19 pandemic in our city. We previously reported the DKA admissions in our COVID-19-free hospital during the pandemic, and these patients were found to be older with a higher proportion of newly diagnosed diabetes and T2DM, and the majority were non-Emirati nationals (Arabs 17.9% vs. 12.7% and South Asian 20.9% vs. 3.8%) (20) when compared to prior to the pandemic (21). Additionally, we identified a small number of T2DM patients with DKA related to SGLT2i, which is an increasingly recognized risk with SGLT2 inhibitor use (22). In the 10 DKA cases of T2DM patients who were using SGLT2 inhibitors, 6 had concomitant infections, 1 had insulin omission, and there were no other concomitant risks in 3 patients. There were no T1DM patients using SGLT2 inhibitors.

Sixteen percent of all global diabetes-related fatalities are due to DKA (3). Our in-hospital mortality rate for patients with T2DM was 6.3%, while the overall mortality rate was 1.8%. Interestingly, all the patients with T2DM and DKA who died were classified as having moderate DKA. The overall mortality rates varied across different regions of the world, with some documenting lower rates than those in our study (23, 24) and others reporting higher rates (25, 26). Although speculative, the factors likely associated with the increased risk of mortality in our patients with T2DM when compared to those with T1DM are older age, the presence of comorbidities (macrovascular complications and CKD), high ICU admission rates, long lengths of stay, long times to recovery, and extended mechanical ventilation needs. Similar results were obtained from a study in Pakistan reporting higher mortality in older patients with concomitant comorbidities and T2DM (24). Interestingly, the presence of severe comorbidities has been found to be a significant independent predictor for mortality in patients with DKA (25, 27). These factors along with the fact that the moderate DKA group constitutes the majority of DKA cases in T2DM (64.6%) in our cohort could explain the high mortality with the moderate DKA. These findings highlight the importance of vigilant glycemic control monitoring in older patients presenting with T2DM and underlying comorbidities.

With regard to the severity of DKA, most of our DKA admissions were classified as moderate (50.9%) with severity differences noted according to sex and disease duration. Almost 84% of the patients in our cohort were admitted to the ICU. Despite this finding being in concordance with a study by Rashid et al. (21), as expected, other studies showed higher rates for ICU admissions for patients with severe DKA (25, 26). A previous study suggests that classifying DKA correlates with the duration of in-hospital stay, requirement of ICU care, and mortality, and is thus a valuable tool to predict outcomes (23). In our cohort, the severe DKA cases were associated with longer time to DKA resolution with no difference in length of hospital stay and complication compared to the moderate DKA group. Additionally, there was no difference between the DKA severity groups in regard to in-hospital mortality and the need for mechanical ventilation. These could be related to several factors including hospital-specific indications for ICU admission in DKA cases, patient’s age, and the presence of concomitant comorbidities.

## Limitations

We are aware of the limitations of this study. This was a retrospective observational study limited to patients selected on the basis of electronic medical records from a single-center hospital. Thus, generalizations to the general population should be approached with caution. A study on the pre-pandemic DKA episodes of patients in the other public facility would probably yield comparatively interesting results, given the differences in the cohorts of patients attending the two facilities.

## Conclusion

The main finding of this study is that patients with uncontrolled DM are at risk of being hospitalized with DKA irrespective of their DM type. T1DM are at a higher risk for DKA but those with T2DM are older with more comorbidities and have a higher mortality. Among Emirati patients with DKA, most patients had T1DM, whereas T2DM was more common among non-Emirati patients with DKA, which highlights the ethnic difference in DKA risk with T2DM. SGLT2 inhibitor use in T2DM is a newly contributing factor to DKA in the recent years that needs to be identified especially with expanding indication for non-glycemic use. DKA is a life-threatening but avoidable complication where complications are higher in moderate and severe groups in our cohort. Therefore, physicians and patients should strive towards optimal long-term DM management by taking preventive measures such as self-management education and education regarding DKA risk in both DM types mainly in ethnicities with higher risk and in SGLT2 inhibitor users.

## Data availability statement

The original contributions presented in the study are included in the article/supplementary material. Further inquiries can be directed to the corresponding author.

## Ethics statement

The studies involving human participants were reviewed and approved by the Tawam Human Research Ethics Committee (MF2058-2022-826). Written informed consent was waived due to the retrospective nature of the study, the anonymity of the data collection, and the lack of interventions.

## Author contributions

All authors were involved in the data collection and the manuscript drafting and finalizing. RA, AS and MA collected the data. SA-S performed the statistical analysis. RA, SA-S and RG provided intellectual input and wrote the manuscript. All the authors reviewed the final draft and have accepted responsibility for its entire content and approved its submission

## Conflict of interest

The authors declare that the research was conducted in the absence of any commercial or financial relationships that could be construed as a potential conflict of interest.

## Publisher’s note

All claims expressed in this article are solely those of the authors and do not necessarily represent those of their affiliated organizations, or those of the publisher, the editors and the reviewers. Any product that may be evaluated in this article, or claim that may be made by its manufacturer, is not guaranteed or endorsed by the publisher.
